# Towards Wind Vector and Wave Height Retrievals Over Inland Waters Using CYGNSS

**DOI:** 10.1029/2020EA001506

**Published:** 2021-07-09

**Authors:** Eric Loria, Andrew O'Brien, Valery Zavorotny, Cinzia Zuffada

**Affiliations:** ^1^ Jet Propulsion Laboratory California Institute of Technology Pasadena CA USA; ^2^ The Ohio State University Columbus OH USA; ^3^ CIRES/University of Colorado Boulder Boulder CO USA

## Abstract

GNSS Reflectometry (GNSS‐R) measurements over inland water bodies, such as lakes, rivers, and wetlands exhibit strong coherent signals. The strength of the coherent reflections is highly sensitive to small‐scale surface roughness. For inland waters, this roughness is primarily due to wind‐driven surface waves. The sensitivity of the coherent reflections to surface roughness can be leveraged to estimate wave height profiles across inland waters. Coupled with a wind wave model, an approach to retrieve a wind vector is described using a forward model, which is potentially able to predict scattered power profiles for different wind speeds and directions and choosing the minimum‐squared error solution. The ability for spaceborne or airborne GNSS‐R to measure an inland water wind vector and wave heights could contribute to scientific applications focused on understanding nearshore ecosystems, monitoring climate change effects on inland waters, sediment resuspension, biomass production, fish habitat, and others. This paper presents a novel approach to potentially retrieve wind vector and wave heights over inland waters using GNSS‐R and discusses the issues with performing such retrievals using simulation and very few available raw signals recorded from CYGNSS satellites.

## Key Points

- Global Navigation Satellite Systems (GNSS) signals reflected from inland waters exhibit coherent scattering properties that make them highly sensitive to water surface roughness
- Wind‐induced roughness will vary across a water body, with a strong dependence on wind speed, wind direction, and the water depth
- Discuss potentials of a novel model‐based approach for retrieval a of wind vector and surface wave heights over lakes using CYGNSS data

## 1 Introduction

Global Navigation Satellite Systems Reflectometry (GNSS‐R) is a remote sensing technique that uses reflected GNSS signals to make measurements of geophysical properties of the Earth's surface. The significant roughness of typical ocean and land surfaces at L‐band frequencies commonly results in diffusely scattered GNSS reflections. However, it has been observed that the surfaces of lakes, rivers, wetlands, and other inland water bodies are often sufficiently smooth to produce coherent reflections. Coherent reflections are of particular interest for remote sensing due to their high‐reflected power, fine spatial resolution, and phase information. Utilizing these reflections enables a unique capability to monitor surface water (even obstructed by dense vegetation) in all weather conditions including rain and clouds. The NASA CYGNSS mission is a constellation of 8 small satellites in low Earth orbit that carry GNSS‐R receivers (Ruf et al., 2013). Each receiver measures GPS L1 C/A‐coded signals as they reflect off the Earth's surface. Coherent reflections have been widely observed in GNSS‐R from both aircraft and spaceborne receivers over inland water bodies since the earliest GNSS reflectometry campaigns, but more recent research has focused specifically on their characterization (Chew et al., 2016, 2017; Morris et al., 2019; Nghiem et al., 2017; Zuffada et al., 2016).

The main scattering surface parameter that determines the strength of a coherent reflection is the electromagnetic surface roughness. For inland water bodies without significant flow, it is expected that surface roughness be primarily due to wind driven waves. While other sources of roughness are possible, such as rain and human activity, these effects are likely limited. Unlike the open ocean, wind driven waves over an inland water body will have their heights limited by a number of factors, including water boundary shape, depth, and fetch. The wave heights will also vary over the water surface. Since coherent reflections in GNSS‐R are very sensitive to small changes in surface roughness, it is feasible that one could measure the changes in scattered power as the minimum path reflection point, called the specular point (the geometric center of the reflection), traverses a water body and infer the wind speed and wind direction (i.e., wind vector) based on this profile. To achieve this, it is necessary to develop a forward scattering and inland water wave model to describe the interaction between wind and waves.

Measurements of the wave heights in inland waters by GNSS‐R are relevant for applications involving estimation of hydrological and biological processes such as sediment resuspension (Hofmann et al., 2011), algae production (Strand & Weisner, 1996), the habitat choice of fish, (Stoll, Hofmann, & Fischer, 2010) and the survival rates of their eggs (Stoll, Probst, et al., 2010), as well as the stress imposed on organisms living near the shoreline (Scheifhacken et al., 2007). In addition, the wave exposure (a description of how much a shoreline is subjected to large waves) can help determine the distribution of vegetation in a water body (van de Koppel et al., 2007). Currently, there is a gap in the measurement of wave heights in lakes at a global scale.

In addition, measurements of the inland wind vector can be used for weather monitoring and forecasting, wind‐load climatology, estimation of air pollution dispersion, estimation of surface fluxes, agricultural applications, and monitoring the effects of climate change on inland waters (World Meteorological Organization (WMO), 2014). The impacts of climate change on the world's lakes are still open questions, although what is known for the largest lake, for example, Lake Superior in North America, is a cause for concern. Over the past decades, mean surface water temperatures in Lake Superior have warmed faster than air temperature during the thermally stratified summer season because decreasing ice cover has led to increased heat input. Desai et al. 2009 found that increasing temperatures in both air and surface water, and a reduction in the temperature gradient between air and water are destabilizing the atmospheric surface layer above the lake (Desai et al., 2009). As a result, surface wind speeds above Lake Superior have been perceptibly increasing at decadal time scales, exceeding trends in wind speed over land. This is significant because changing wind speeds can lead to changes in the currents. Changes in the currents affect mixing, with possible repercussions on the atmospheric circulation and biogenic cycle of the lake and its surroundings. By contrast, Woolway et al. 2017 analyzed the large shallow Lake Võrtsjärv, where substantial changes in thermal stratification dynamics are attributed to decreasing wind speeds, consistent with the globally observed atmospheric stilling, a decrease of wind speed that has been occurring globally over land for more than three decades.

While the surface temperature and associated trends of inland water bodies can be accurately measured (for clear sky conditions) with thermal infrared data from satellite instruments (Schneider & Hook, 2010), it is more difficult to measure winds, and global datasets for lake surface temperature and winds are lacking (particularly moderate and small size lakes) since very few lakes are instrumented. Such data would be very valuable to investigate lake evaporation and the larger context of the lake surface heat balance.

Furthermore, accurate modeling of wind driven surface roughness will allow this effect to be removed from GNSS‐R observations of inland water bodies, revealing other features to be measured, such as change in surface water extent or vegetation types and densities.

This paper builds upon the previous work in (Loria, O'Brien, Zavorotny, & Zuffada, 2020) and presents a novel GNSS‐R method using the variations in coherent reflected power from inland waters to discuss a potential retrieval of both the wind vector and wave heights. Section 2 provides an overview of the conditions that lead to coherence and the importance of small‐scale surface roughness. Section 3 presents a wind‐driven wave model for inland water bodies that is significantly different from wave models used for GNSS‐R in open ocean scenarios. Section 4 applies the wave model and coherent scattering model to an example lake. CYGNSS raw signal measurements are compared to simulated lake conditions and used to retrieve the wind speed. Simulated results not only show that there is significant sensitivity to the wind vector, but comparisons to nearby anemometer readings confirm accurate retrieval of wind values.

## 2 Coherence and Small‐Scale Surface Roughness

One of the most significant parameters that determine the magnitude of the coherent return is the surface roughness. The amount of height variation across a surface–relative to the incidence angle and wavelength–will determine the amount of power that is coherently reflected. The relative electromagnetic roughness is typically described by the Rayleigh parameter (Beckmann & Spizzichino, 1963)

(1) $$R_{a}=2\pi {\langle \Delta h^{2}\rangle }^{\frac{1}{2}}\frac{cos\theta }{\lambda },$$

where Δ*h* describes the standard deviation of the surface height distribution, *λ* is the wavelength, and *θ* is the incidence angle of the reflection. While this treatment of surface heights applies to any Gaussian surface, this work is focused on examining the reflections from inland waters. For water surfaces, it is common to describe the surface wave height in terms of significant wave height (*H*_*s*_), and the Rayleigh parameter *R*_*a*_ can be rewritten in terms of *H*_*s*_ as

(2) $$R_{a}=0.5\;\pi H_{s}\;\frac{cos\theta }{\lambda }.$$

The loss in the coherence of the scattered fields with increased roughness *R*_*a*_ leads to a reduction in the coherent signal amplitude. The attenuation in the coherent signal power due to surface roughness (*ψ*) is given by (Ulaby et al., 2014)

(3) $$\psi =e^{-4R_{a}^{2}}.$$

**Figure 1:**
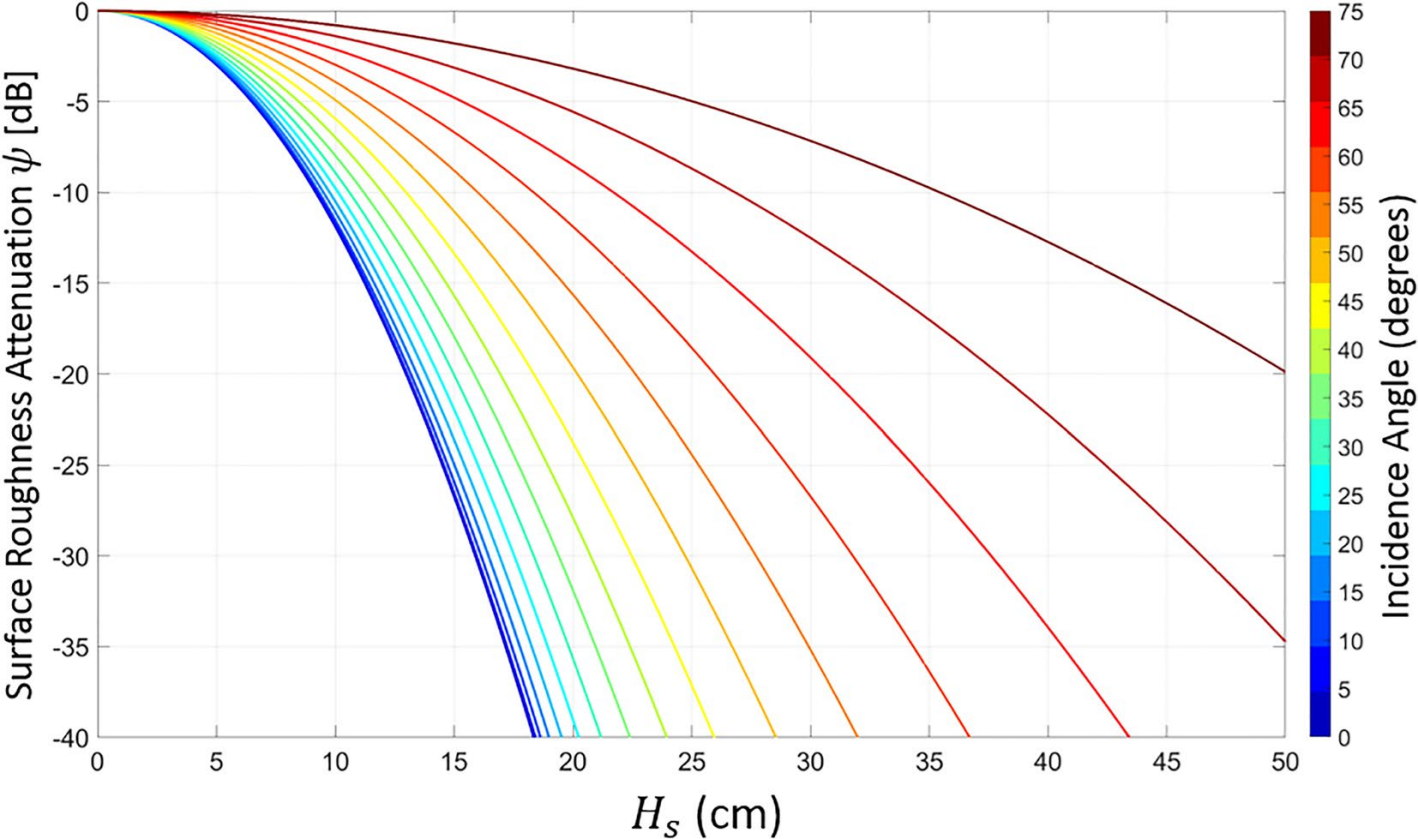
Attenuation of the reflected coherent power at 1,575 MHz for increasing *H*_*s*_ at varying incidence angles.

Figure 1 shows the increasing attenuation of the coherent signal for increasing *H*_*s*_ at the GPS L1 frequency (1,575 MHz). Lower frequencies, such as L2 (1,227 MHz) or L5 (1,176 MHz), will have slightly lower attenuation for the same wave heights, given their larger wavelengths. Incidence angle (shown by the color of the curves) will also affect the total loss through Equation 2. For GNSS reflections, the coherent component generally exists for *H*_*s*_ < 15–20 cm. Also of note is that at lower incidence angles, increasing roughness will attenuate signals more quickly.

(4) $$\langle {\left|Y\left(\tau ,f\right)\right|}^{2}\rangle =\frac{\lambda^{2}P_{t}G_{t}}{{\left(4\pi \right)}^{3}}\iint \frac{G_{r}{\left|\chi \left(f,\tau \right)\right|}^{2}}{R_{1}^{2}R_{2}^{2}}\gamma \sigma_{pq}^{0}dS,$$

The general form of the power of the bistatic signal measured by a GNSS‐R receiver can be written as (Zavorotny & Voronovich, 2000)where *λ* is the wavelength, *P*_*t*_ is the transmit power, *G*_*r*_, *G*_*t*_ are the receiver and transmitter antenna gains, *χ*(*f*, *τ*) is the Woodward Ambiguity Function (WAF), *R*_1_ and *R*_2_ are the distances from transmitter to a point on the surface, and from the surface to the receiver respectively, *γ* is a vegetation attenuation parameter, *dS* is a differential surface area, and $\sigma_{pq}^{0}$ is the normalized bistatic radar cross section (NBRCS). The total received power can be split between coherent and non‐coherent components (see, e.g., [Alonso‐Arroyo et al., 2015; Voronovich & Zavorotny, 2018; Zavorotny et al., 2014]). While the non‐coherent component is the dominant scattering mechanism for most Earth surfaces, areas that exhibit low surface roughness (given by a low Rayleigh parameter *R*_*a*_), produce a coherent component.

The power of the coherently reflected signal is often approximated by assuming a large plane surface covered by a small‐scale roughness. In this case, the coherent component of the power can be written as (Fung & Eom, 1983, Ulaby et al., 2014)

(5) $$\langle {\left|Y_{coh}\left(\tau ,f\right)\right|}^{2}\rangle =\frac{P_{t}G_{t}}{4\pi {\left(R_{t}+R_{r}\right)}^{2}}\frac{\lambda^{2}G_{r}}{4\pi }{\left|\chi \left(\tau ,f\right)\right|}^{2}\gamma {\left|\Gamma \right|}^{2}\psi .$$

This is an analog of Friis transmission formula assuming the reflection obeys image theory. This equation only applies when the scattering surface is sufficiently large and homogeneous. This assumption of homogeneity also applies to the surface roughness, and thus the Rayleigh parameter *R*_*a*_. However, this homogeneity does not occur in nature over water bodies. Different areas of inland water bodies will typically have different amounts of surface roughness. Each area will contribute differently to the total received power. Thus, we move to the use of a surface integral that can handle the heterogeneity in a given scene (Loria, O'Brien, Zavorotny, Downs, & Zuffada, 2020),

(6) $$\langle {\left|Y\left(\tau ,f\right)\right|}^{2}\rangle =\frac{G_{r}G_{t}P_{t}}{{\left(4\pi \right)}^{3}}{\left|\iint_{S}\frac{jk\sqrt{\gamma \psi }\cos\left(\theta \right)\Gamma \left(\theta \right)\chi \left(f,\tau \right)}{R_{1}R_{2}}e^{-jk\left(R_{1}+R_{2}\right)}dS\right|}^{2},$$

where *k* is the wavenumber and Γ is the Fresnel reflection coefficient. For areas without vegetation (i.e., *γ* = 1), most variables in Equation 6 are well‐known and we are left with two significant sources of variability to consider: The surface geometry *S* and the roughness attenuation *ψ*. For the purpose of this paper we will assume that the inland water geometry *S* is known or well‐observed (i.e., from SAR or optical imaging sensors).

In the next section, we discuss the forward model used to describe the wind/wave interaction and examine how different geophysical parameters such as fetch, wind speed, and wind direction will affect the reflected signal.

## 3 Modeling of Significant Wave Heights Over Inland Waters

To predict the reflected coherent power from an inland water body, it is necessary to first predict the spatially varying surface roughness attenuation *ψ* over the entire surface area *S*. To achieve this, this work applies a water wave model developed by the Coastal Engineering Research Center (CERC) of the U.S. Army Corps of Engineers to calculate surface roughness (Shore Protection Manual, 1984). In the CERC model, the significant wave height is given by

(7) $$H_{s}=\frac{U_{A}^{2}}{g}0.283\tanh\left[0.53{\left(\frac{g\cdot d}{U_{A}^{2}}\right)}^{\frac{3}{4}}\right]\cdot \tanh\left\{\frac{0.00565{\left(\frac{g\cdot F}{U_{A}^{2}}\right)}^{\frac{1}{2}}}{\tanh\left[0.53{\left(\frac{g\cdot d}{U_{A}^{2}}\right)}^{\frac{3}{4}}\right]}\right\}.$$

In this equation, $U_{A}=0.71U_{10}^{\left(1.23\right)}$ is the wind stress factor, *g* is the gravitational acceleration $\left(m/s^{2}\right)$, *d* is the water depth (m), and *F* is the fetch (m). The variable *U*_10_ is the wind speed (m/s). It is important to note that there are three main factors affecting the wave heights in this model. They are *U*_10_, *d*, and *F*. In the context of this model, fetch is defined as the distance from a given location to the shoreline in the up‐wind direction. This model was developed for shallow water conditions, which is considered to be less than 90 meters deep. The model estimates *H*_*s*_, which is then combined with the incidence angle *θ* and wavelength *λ* to form the roughness *R*_*a*_.

The CERC model is a relatively simple wave height model. Other wave models–such as SWAN (Booij et al., 1996; Jin & Ji, 2001) – are numerically calculated utilizing more information about the wind, bottom friction, and water currents. These models also consider the diffraction and reflection of waves from water boundaries, unlike the CERC model. While more accurate models such as SWAN can be used to produce more realistic descriptions of the significant wave heights, they may be too computationally expensive or may require more information than is generally known about a given inland water body. In addition, the CERC wave model was shown to be accurate for use in lakes (Seibt et al., 2013). Since this work is focused on examining the sensitivity of the strength of the coherently reflected GNSS signals to general surface wave height variation over the surface, the CERC model is sufficient.

The next few sections describe how each input to the CERC model affects the calculated wave heights.

### 3.1 Wind Speed

**Figure 2:**
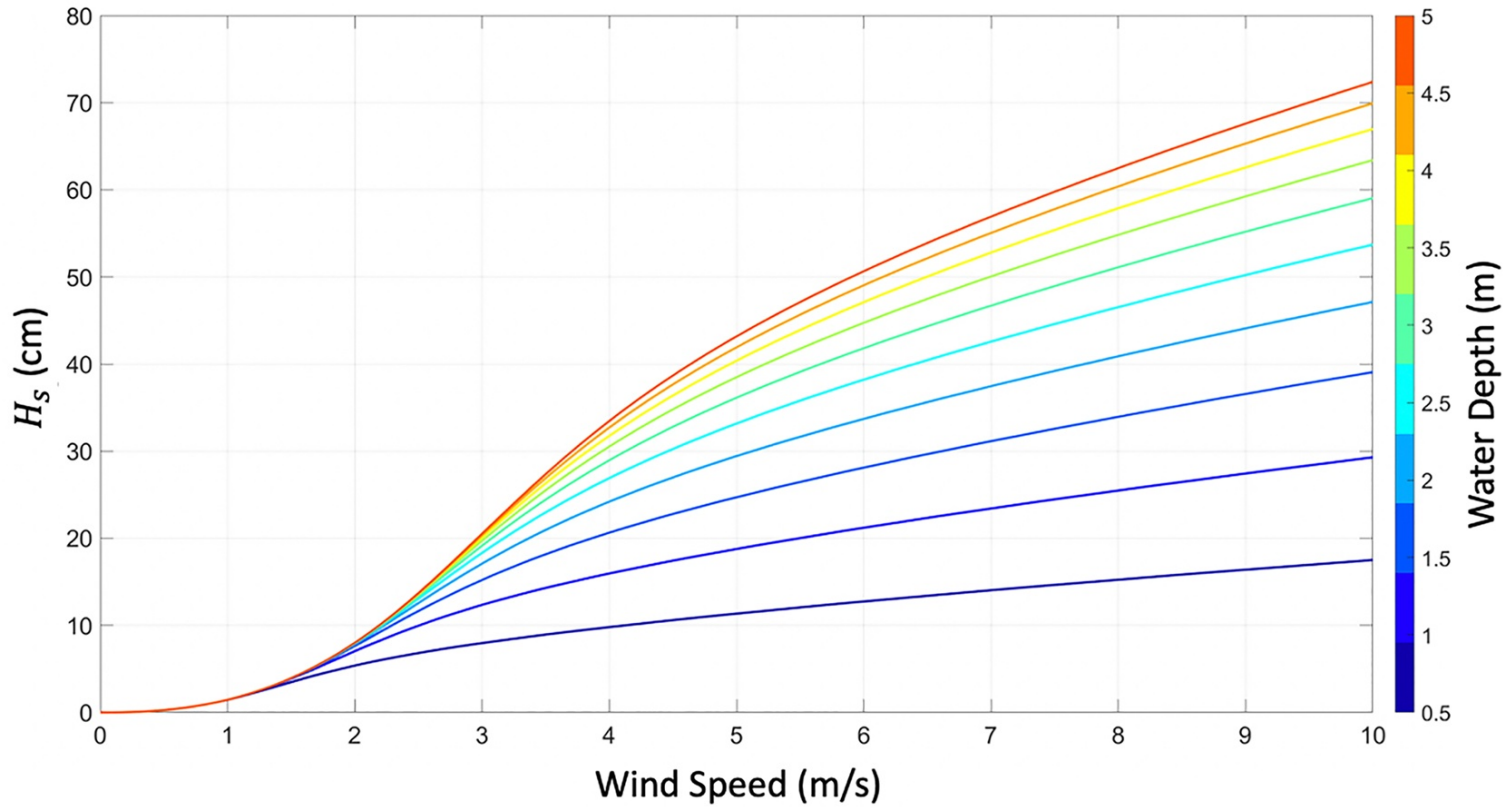
*H*_*s*_ for increasing wind speeds, at several water depths (m), shown by varying color curves. Fetch is fixed at 2 km.

Water waves accounted for in this model come from the interaction between wind and the water surface. Friction between the wind and the water surface generates these surface waves (Fontaine, 2013). As wind speed increases, the size of the waves will generally also increase. An example of significant wave height driven by winds as described by the model Equation 7 is shown in Figure 2. In this simplified model for the wind‐driven waves, the wind is approximated as being constant in magnitude and uniform in the direction. That is, over the size of the surface we are evaluating (approximately 10 × 10 km), the wind field is assumed homogeneous. More complex numerical modeling techniques could be used to calculate the surface wave height profiles using heterogeneous wind conditions, but that is outside the scope of this work.

### 3.2 Water Depth

**Figure 3:**
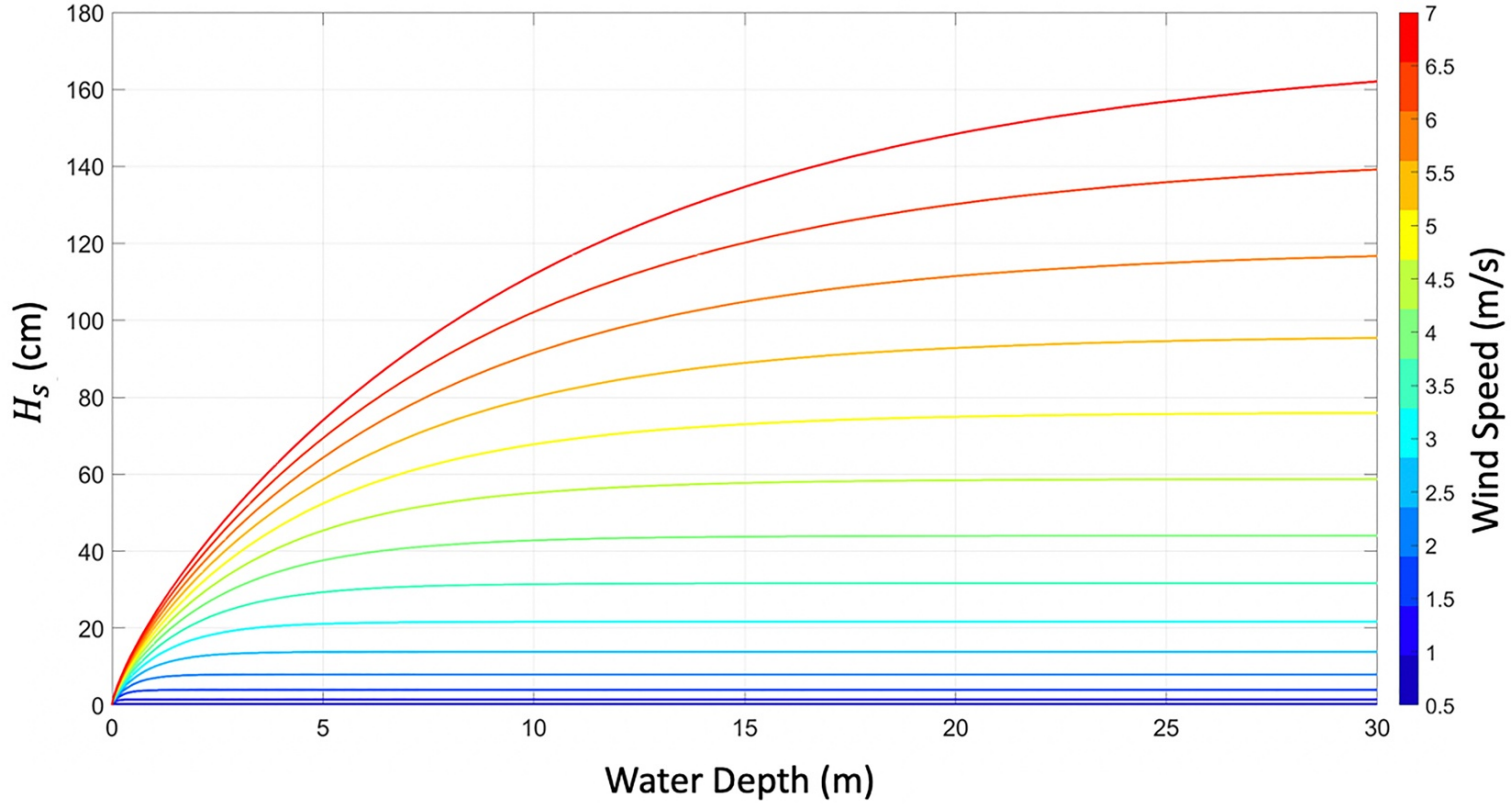
*H*_*s*_ for increasing water depth at various wind speeds (m/s), indicated by the colored curves.

The size of waves on the surface can be constrained by the depth of the water. Friction between the water and the floor of the water body will create a cap on the size of the waves. This is modeled in Equation 7 through the hyperbolic tangents, and the results can be seen in Figure 3. The wave heights were generated using a large value of fetch (20 km) so that it would not be the limiting factor. As the wind speed increases, *H*_*s*_ increases until it reaches a maximum that is limited by the depth.

### 3.3 Fetch

**Figure 4:**
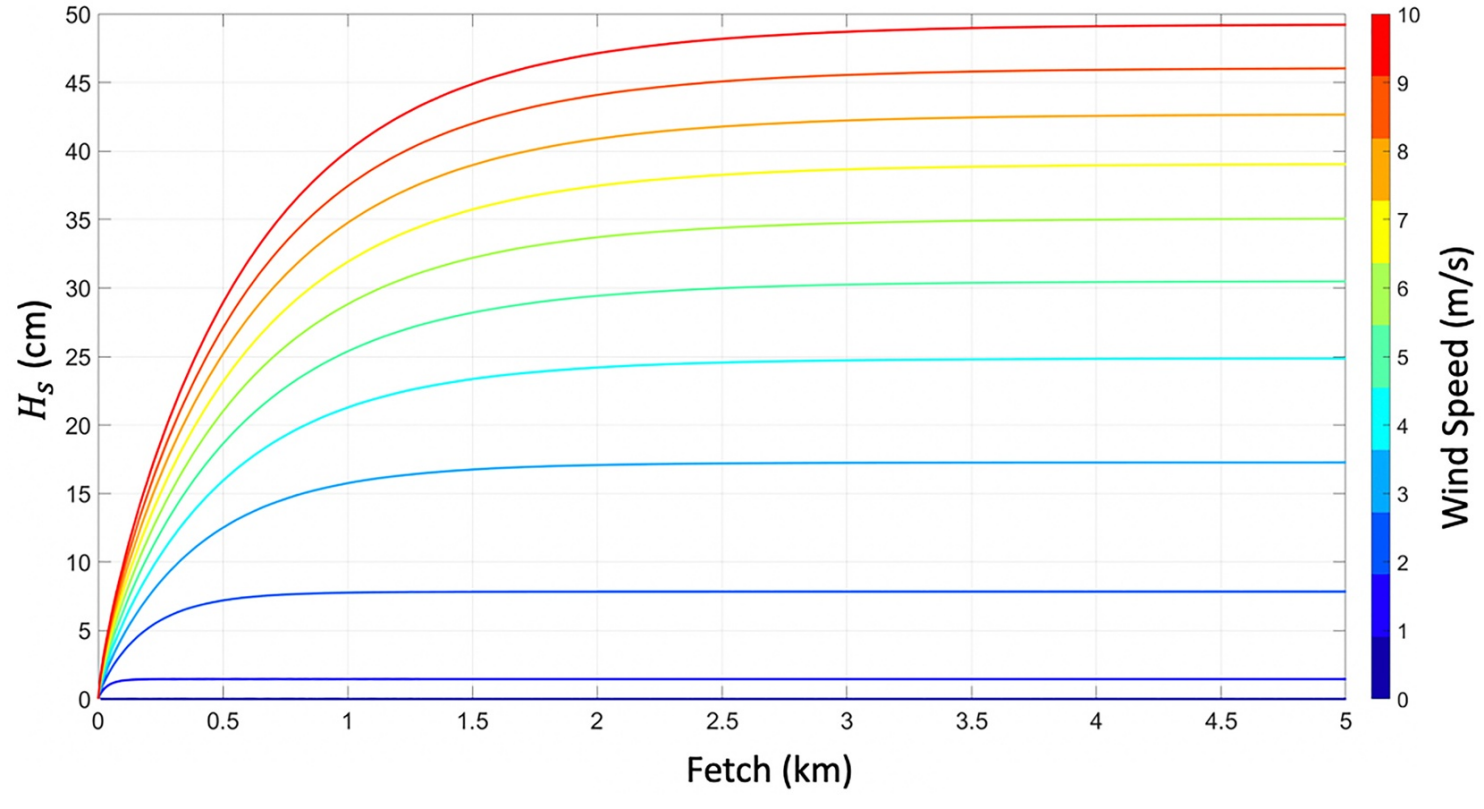
Significant wave height *H*_*s*_ for increasing fetch at various wind speeds. Water depth is fixed at 2 m.

Fetch is another factor in the model that affects the surface wave heights. As wind blows over the water surface, waves are generated and grow with time and space (Fontaine, 2013). Fetch is the distance from a given location on the water body to the coastline in the up‐wind direction. In a manner very similar to water depth, small fetch will constrain the maximum size of the waves. Figure 4 shows an example of *H*_*s*_ for increasing fetch. It can again be seen that increased fetch allows for larger waves for a given wind speed. Eventually, either the fetch or the water depth will constrain the maximum wave size for a particular wind speed.

It is through fetch that we see the received power's sensitivity to wind direction. The value of fetch is calculated from a point on the surface to the nearest shoreline in the up‐wind direction. This means that as wind direction changes, the value of fetch at a given point may vary with the boundary of the inland water body. Subsequently, the modeled wave heights predicted by Equation 7 may vary as well. While the definition of fetch is simple, calculating fetch across a water body numerically can be challenging. First, for a given water body, the shorelines must be found using pre‐existing water masks. In a binary watermask (given as a “1” or a “0”), the transition between the two values would represent the shoreline. Then, for the given point on the surface that the fetch is being estimated for, a line is drawn between every shoreline point in the contour curve and the given point on the surface. The angles (in the same sense as the wind direction) of all of these lines are then calculated and compared to the up‐wind direction. The length of the line whose angle most closely matches the up‐wind direction is the estimated fetch. The total number of fetch lines calculated for each point within the water body is relative to both the resolution of the watermask and the size of the water body itself.

## 4 Example Results

**Figure 5:**
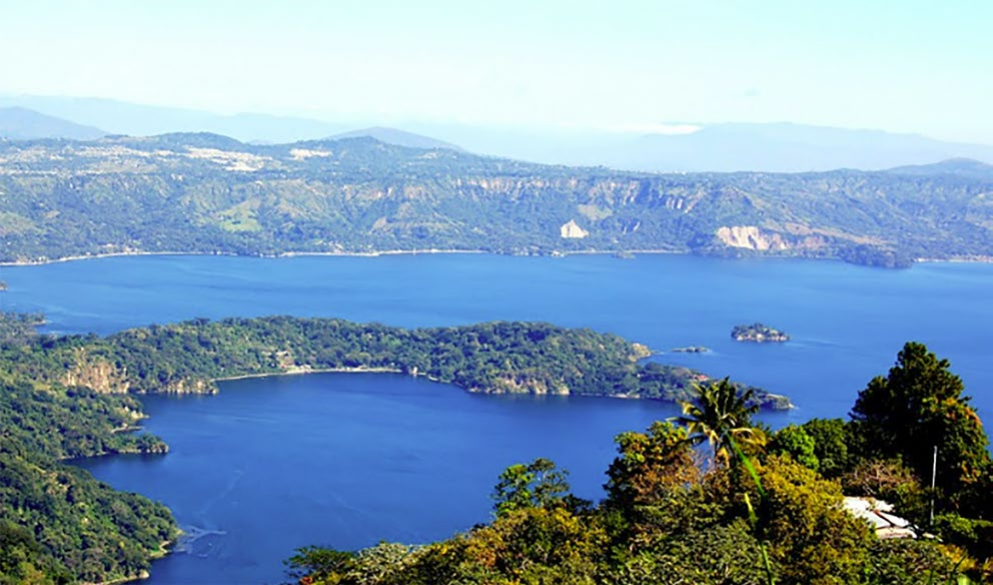
Photograph of Lake Ilopango in El Salvador (Diaz, 2015).

Lake Ilopango in El Salvador was chosen as a target to test the wind/wave model and wind vector retrieval using our scattering model. This lake was chosen because it has a stable shape, does not change significantly with time, and is within the latitude range of CYGNSS. Figure 5 shows a photo of the lake. It is a crater lake, approximately eight × 11 km in size and surrounded by rough, mountainous terrain. The lake itself is also isolated from other inland waters. Due to the rough terrain, no significant contributions to the total reflected signal from land is expected. This permits us to approximate the reflected signal power as originating solely from the water surface in our model. For applications of the proposed retrieval approach to other areas where the land is very flat, coherent reflections may be possible and should be considered.

### 4.1 Modeling Surface Roughness for Lake Ilopango

**Figure 6:**
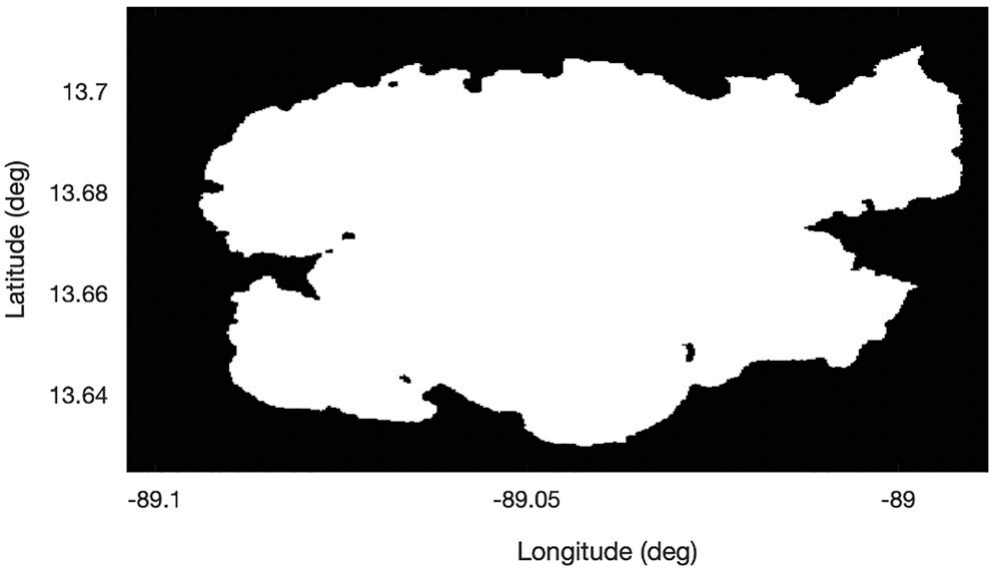
Water mask from (Pekel et al., 2016) for Lake Ilopango where white indicates water and black indicates land.

A water mask from (Pekel et al., 2016) was obtained for Lake Ilopango. Figure 6 shows a plot of the watermask data. The general watermask data were converted into a binary watermask (given as a “1” or a “0”) using the occurrence data field (a description of the frequency where surface water is likely to be present) where any area with above 80% occurrence is given a “1.” However, for this lake with its very stable shape, the choice of the 80% threshold is not important. For other inland waters, selecting an appropriate threshold in order to best match the true shape may be more important.

**Figure 7:**
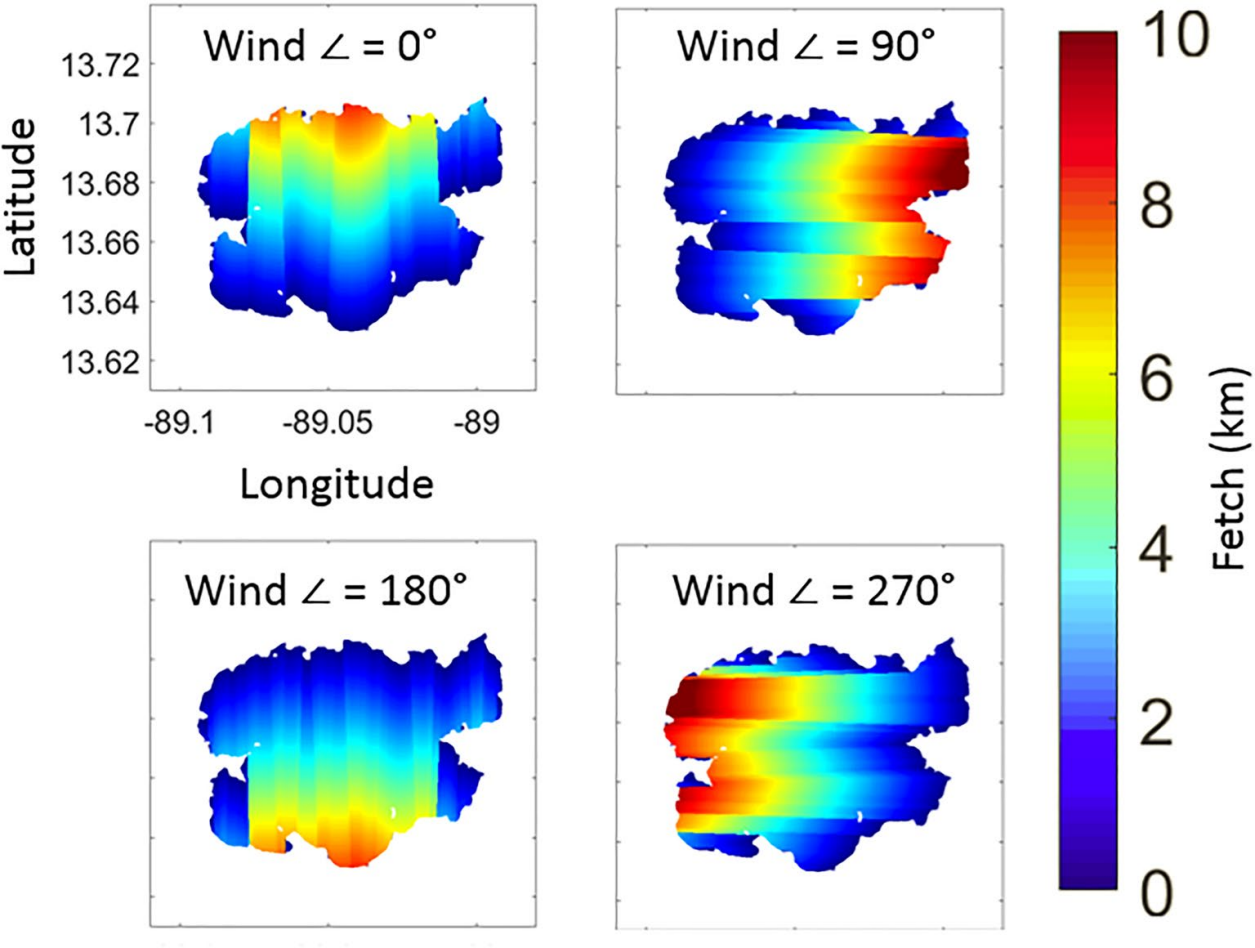
Calculated fetch values across the surface of Lake Ilopango for wind directions of 0°, 90°, 180° and 270°.

Example calculations of fetch across the surface of Lake Ilopango for four wind directions are shown in Figure 7. The wind directions of 0°, 90°, 180°, and 270° correspond to wind originating from the south, west, east, and north directions respectively. It can be seen that areas of the surface with the highest fetch occur where the lake is longest and the wind is blowing in that direction. For example, with a wind direction of 90°, the eastern part of the lake shows much higher fetch than the western part. That is because the distance to the nearest shoreline in the upwind direction, in this case westward, is longest near the eastern shoreline. This is important because different wind directions will result in different surface roughness profiles.

**Figure 8:**
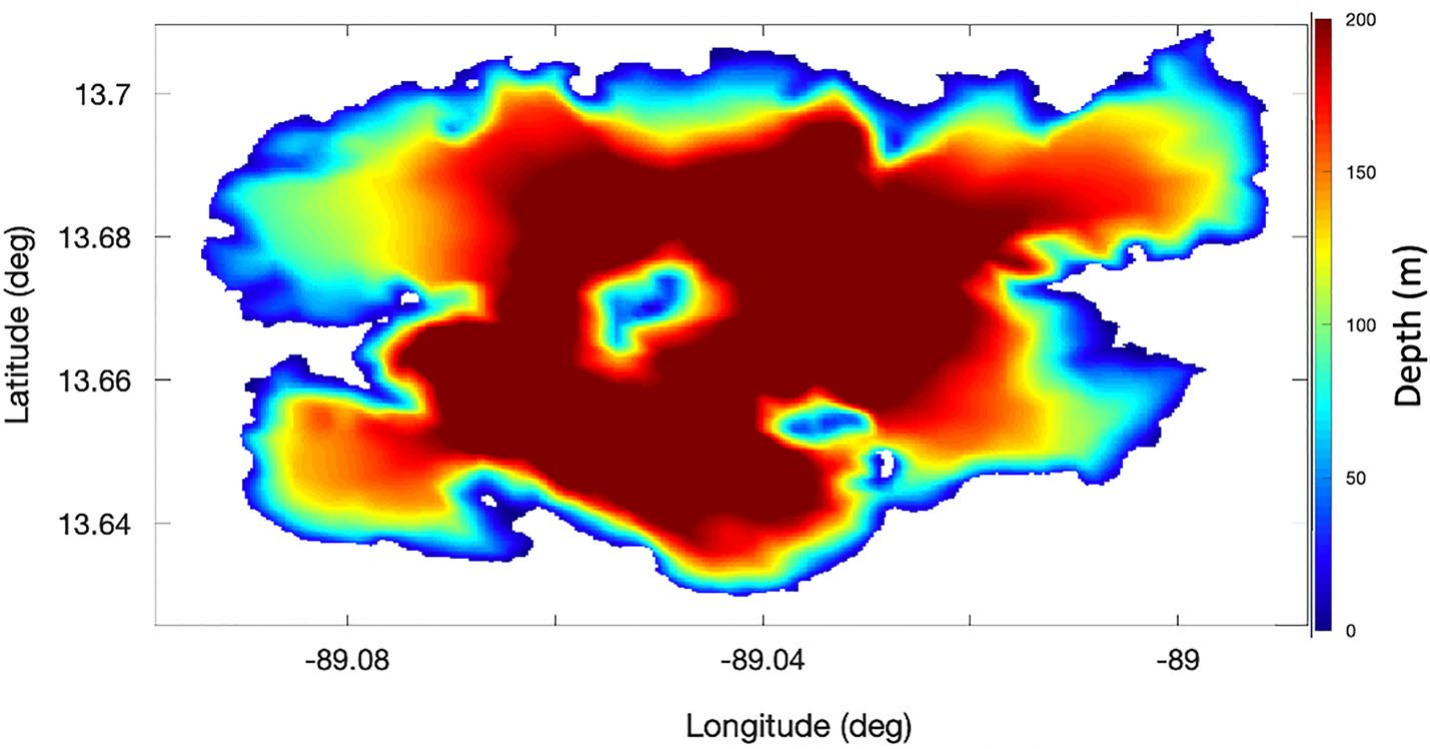
Measurements of Lake Ilopango bathymetry (Gottsmann, 2020).

Along with the wind speed and the fetch across the surface, the third input to Equation 7 is the water depth, *d*. For deeper water bodies, precise knowledge of the bathymetry may not be necessary, as the depth will not be the limiting factor on wave heights. For shallower water bodies, depth can be an important limiter to surface wave height. However, Lake Ilopango is quite deep in many parts. Figure 8 displays the measured lake depth data, which were provided by Dr. Joachim Gottsmann (Gottsmann, 2020) and is described further in (Saxby et al., 2016). Shallower water that may limit wave sizes occurs near the coastlines and is the most relevant to manifestations of strong coherent reflections.

**Figure 9:**
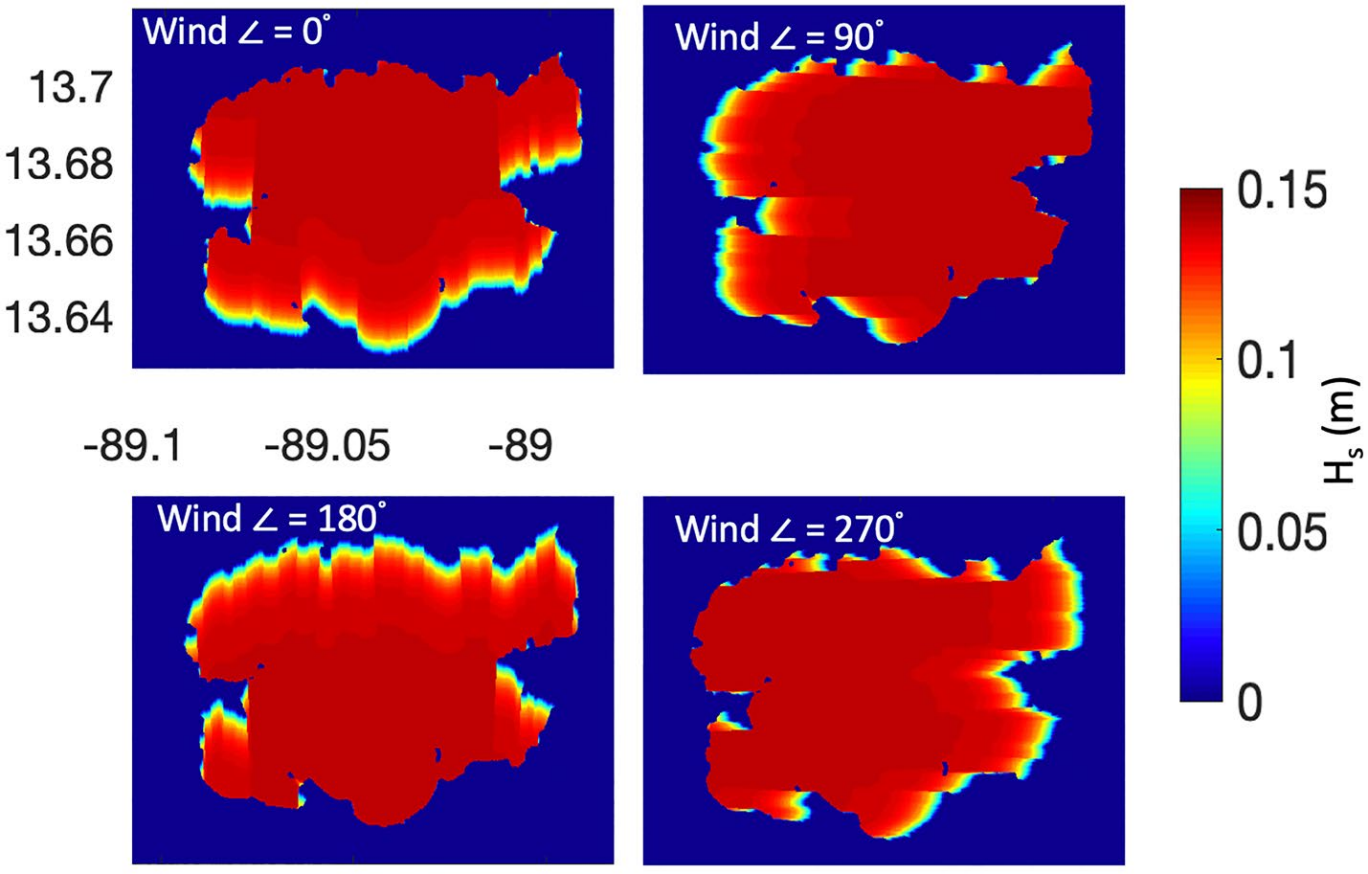
Significant wave height profiles predicted by CERC model at 2.5°m/s for the cardinal wind directions over Lake Ilopango.

As the wind direction changes, the value of fetch across the surface of an inland water body also varies. This change in fetch allows for a change in the wave heights across the surface and a change in the surface roughness. Figure 9 shows an example of calculated significant wave heights across the surface of Lake Ilopango. It can be seen here that for a given wind speed; the wind direction can affect the profile of the roughness across the surface. Generally, the areas of the water body closer to the up‐wind direction will have smaller waves than the areas down‐wind. It is through this variability in the wave heights, and the subsequent change in the surface roughness loss *ψ*, that GNSS‐R measurements from inland waters with coherent scattering may be sensitive to wind direction. This sensitivity will typically be magnified near the shorelines of the water bodies, where the lake will likely either have a maximum or minimum value of fetch. In this way, the profile of coherent scattered power across the inland water body can reveal wind direction information that would otherwise not be present in the individual Delay‐Doppler Map (DDM) measurements. The DDM is the primary measurement made by GNSS‐R receivers, and represent the measured power as a function of varying delay and Doppler values, as seen in Equation 4. The delay and Doppler values correspond to varying areas of the surface around the specular point (the geometric center of the reflection) and is described in more detail in (Zavorotny & Voronovich, 2000).

### 4.2 Sensitivity of CYGNSS SNR to Wind Vector

Generally, the range of wind speeds that the coherent signal will be sensitive to is based on the interaction between the wind and the water surface. Significant wave heights greater than 15–25 cm will greatly attenuate a GPS signal at ∼19 cm wavelength for most incidence angles. At higher incidence angles, the surface roughness attenuation is reduced and leads to coherent reflections that are still strong at higher wave heights. This, in turn, will reduce the sensitivity to lower wind speeds as the effective roughness will be minimal. Generally, the range of wind speeds that a reflection will be sensitive to will be highly dependent on the water body itself (water depth, size, and shape) as well as the reflection geometry.

**Figure 10:**
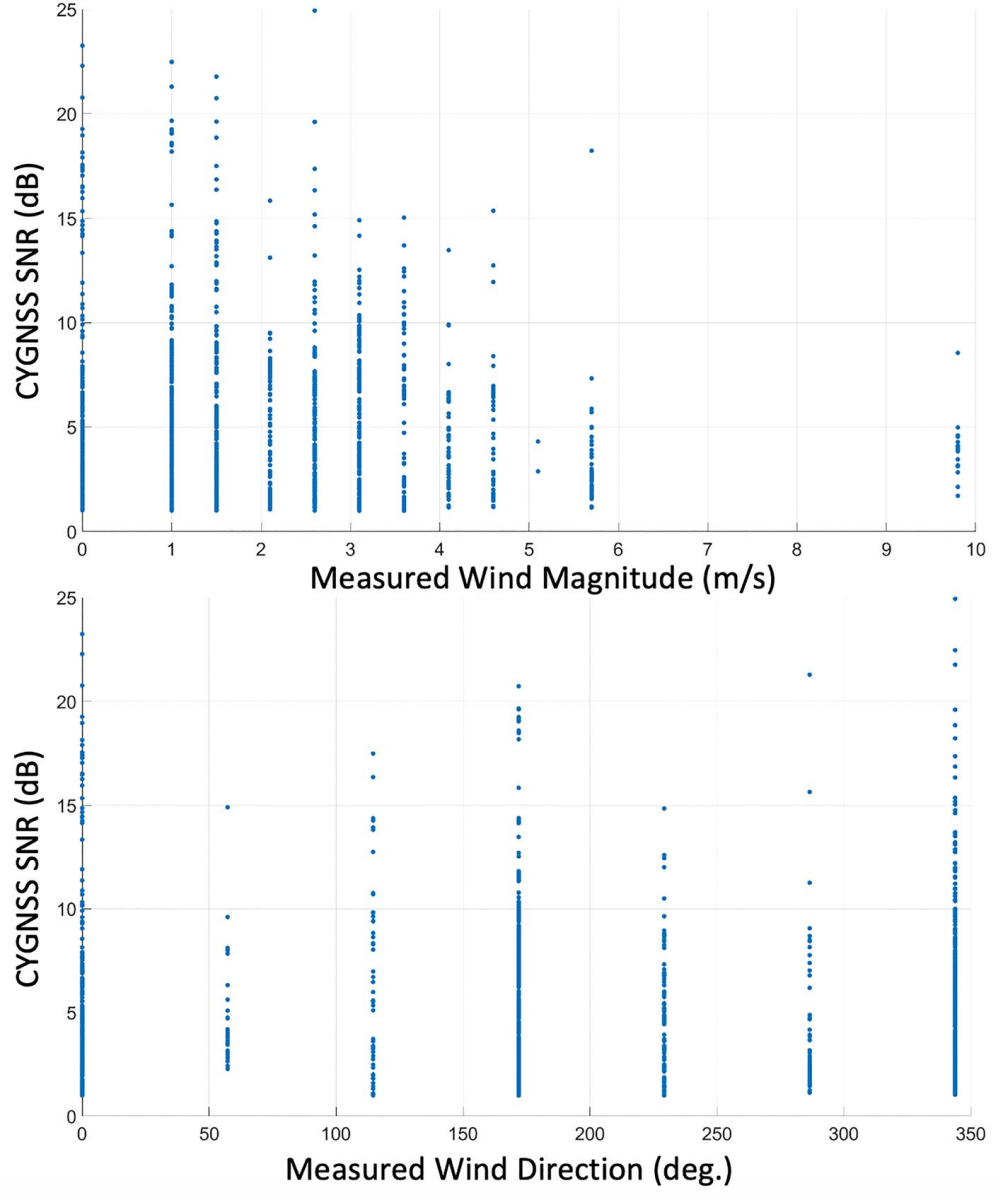
CYGNSS peak SNR versus measured wind speed (top) and wind direction (bottom). Measurements made at Ilopango airport weather station.

Before attempting to retrieve wind speed or wind direction, an analysis was performed to test what sensitivity to wind speed and direction of the DDM peak power is present in the CYGNSS Level‐1 data (CYGNSS, 2017). First, CYGNSS Level‐1 DDMs from Lake Ilopango were collected over an 18 month time frame. Measurements of wind speed and direction are available from a weather station located at the Ilopango airport (∼2.5 km northwest of the lake). The wind measurements from the station are reported every hour, the direction is provided in ∼55° resolution, and the wind speed is provided in ∼0.5 m/s increments (Smith et al., 2011). The SNR reported by CYGNSS v2.1 Level‐1 data of each DDM is matched to the nearest wind measurement in time. CYGNSS SNR values are a peak‐to‐mean ratio of the peak DDM bin power and an average of noise bin values. These estimates of the wind vector and the measured SNR are accumulated and shown in Figure 10 as a scatter plot. The top of Figure 10 shows the SNR versus wind speed. There is a clear pattern in the data where the measured wind speed increases as the measured SNR decreases. This is the expected behavior from the reflected power, as our models predict that the surface roughens with increased wind speed and the total reflected power decreases accordingly. The bottom of Figure 10 shows that the measured SNR's relationship to wind direction is more complicated. Note that the lake is longer in the East‐West direction than the North‐South. Additionally, the small peninsulas on both the East and West shorelines reduce the fetch (on average) in the North‐South direction. For the East‐West wind direction, the fetch is generally higher and the wave heights will be larger. In Figure 10, a general decrease in the accumulated SNR values is observed for reported wind directions more toward the East or West (i.e., 55°, 115°, 230°, 285°) than those reported to be North or South (i.e., 0°, 170°, 340°). This again is consistent with the expected relationship between fetch and wave heights from the model. The measured SNR of DDMs from the CYGNSS Level‐1 data over Lake Ilopango exhibit a clear sensitivity to wind direction. This sensitivity is due to the way the water body supports waves of different sizes based on wind direction for a given wind speed. This wind direction dependence is very different than the one typically assumed over open ocean conditions, where wind direction does not impact wave height. Previous works have also identified the direction‐dependent effect on roughness in GNSS‐R measurements using ground stations in a coastal location (Hoseini et al., 2020). In the next section, the behavior of CYGNSS tracks (measured power over time, as the specular point traverses over a target) over Lake Ilopango are modeled in detail and compared to measurements derived from CYGNSS raw signal data.

### 4.3 Wind Vector and Wave Height Retrieval Using the Forward Model

The sensitivity in the reflected power to the wind speed and direction can be leveraged to make estimations of these two parameters using the modeled wave height profiles. It is expected that, over a certain range of effective roughness (given by the Rayleigh parameter), the coherent signal will be attenuated in unique ways along a particular track across an inland water body. A forward model is used to estimate a set of received power profiles that can be used along with the measured power profile within a least squares error algorithm.

**Figure 11:**
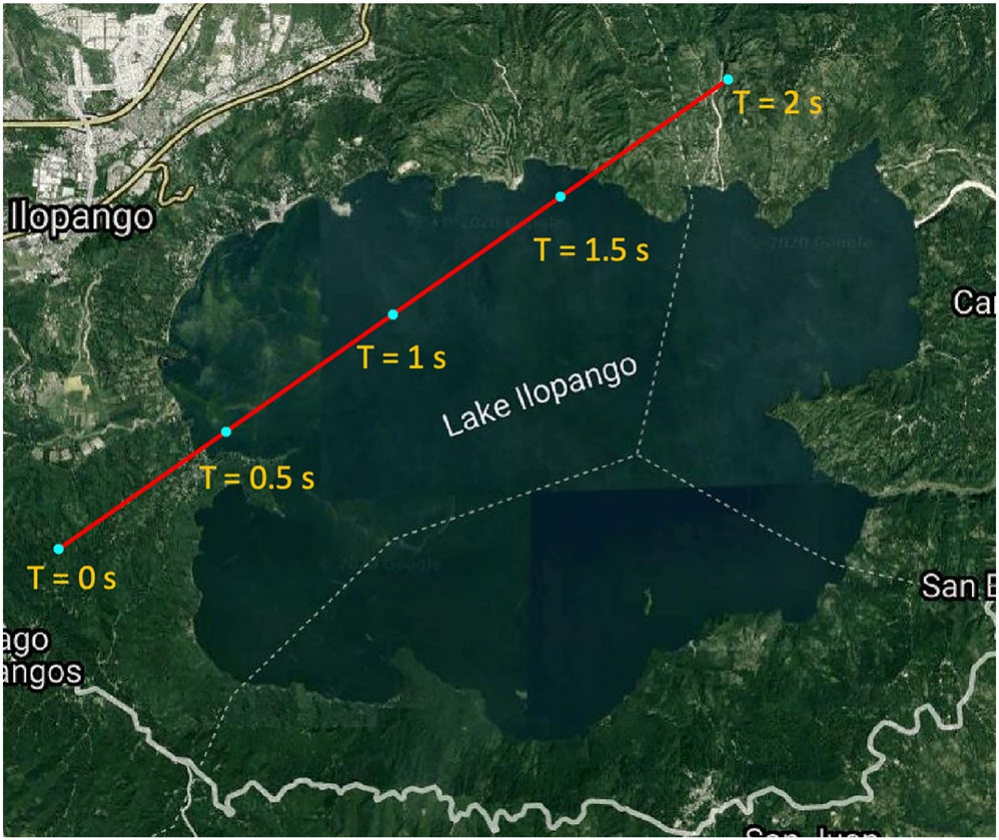
Specular track over Lake Ilopango on July 13, 2019. Specular point moves from southwest to northeast direction. Labeled points indicate corresponding specular locations of the time values used in Figure 15.

For this minimization, a recording of raw samples from CYGNSS taken directly after the analog‐to‐digital converter at an intermediate frequency (called the raw IF mode) is used from a specular track over Lake Ilopango from July 13, 2019, illustrated in Figure 11. The raw samples were processed using a custom GNSS‐R software receiver developed by the authors. The total power received by a satellite is dependent on the roughness profile over the surface. Using the ancillary information about the reflection geometry and scene (antenna gains, effective isotropic radiated power (EIRP), etc.,) a set of power curves is produced for a given track. The power curves are predictions of the power received by the GNSS‐R instrument in Watts for the given reflection scenario. This power is then converted into an SNR using a receiver noise power N and the post‐correlation bandwidth BW (given by BW = 1/*T*_*c*_ where *T*_*c*_ is the coherent integration time) for comparison with the measured data (Gleason et al., 2019). Total receiver noise power is assumed to be a constant value over each track, given by (Gleason et al., 2016)

(8) N=k[(NF−1)290]BW,

where *k* is Boltzmann's constant and NF is the receiver noise figure.

**Figure 12:**
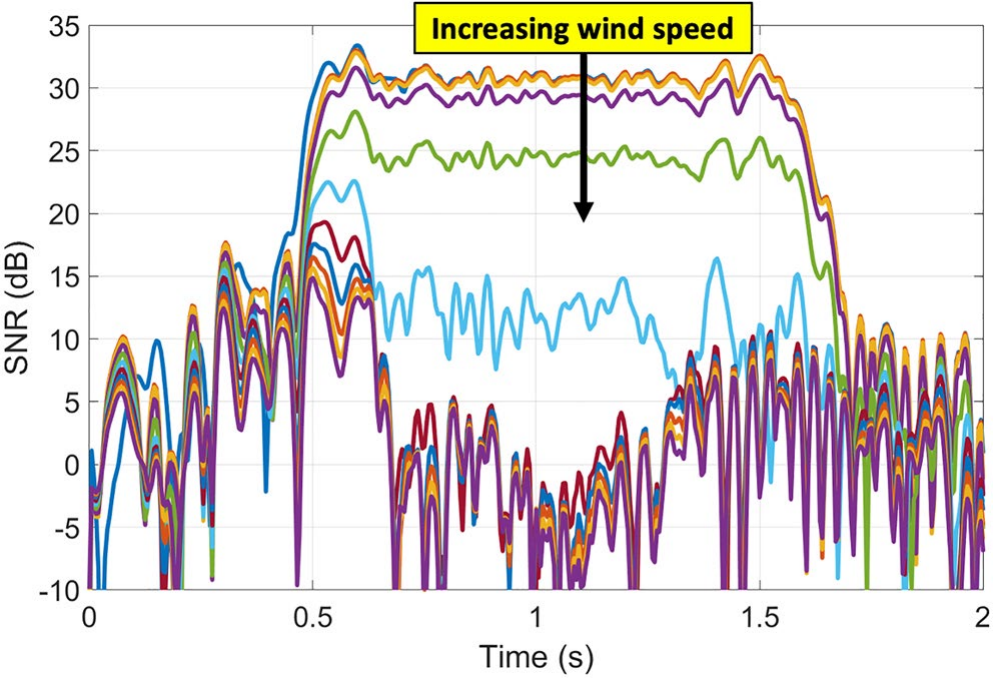
Simulated received power waveforms in terms of SNR (dB) for day 194 track over Lake Ilopango. Wind speed varies from 0 to 5 m/s, wind direction toward NE.

**Figure 13:**
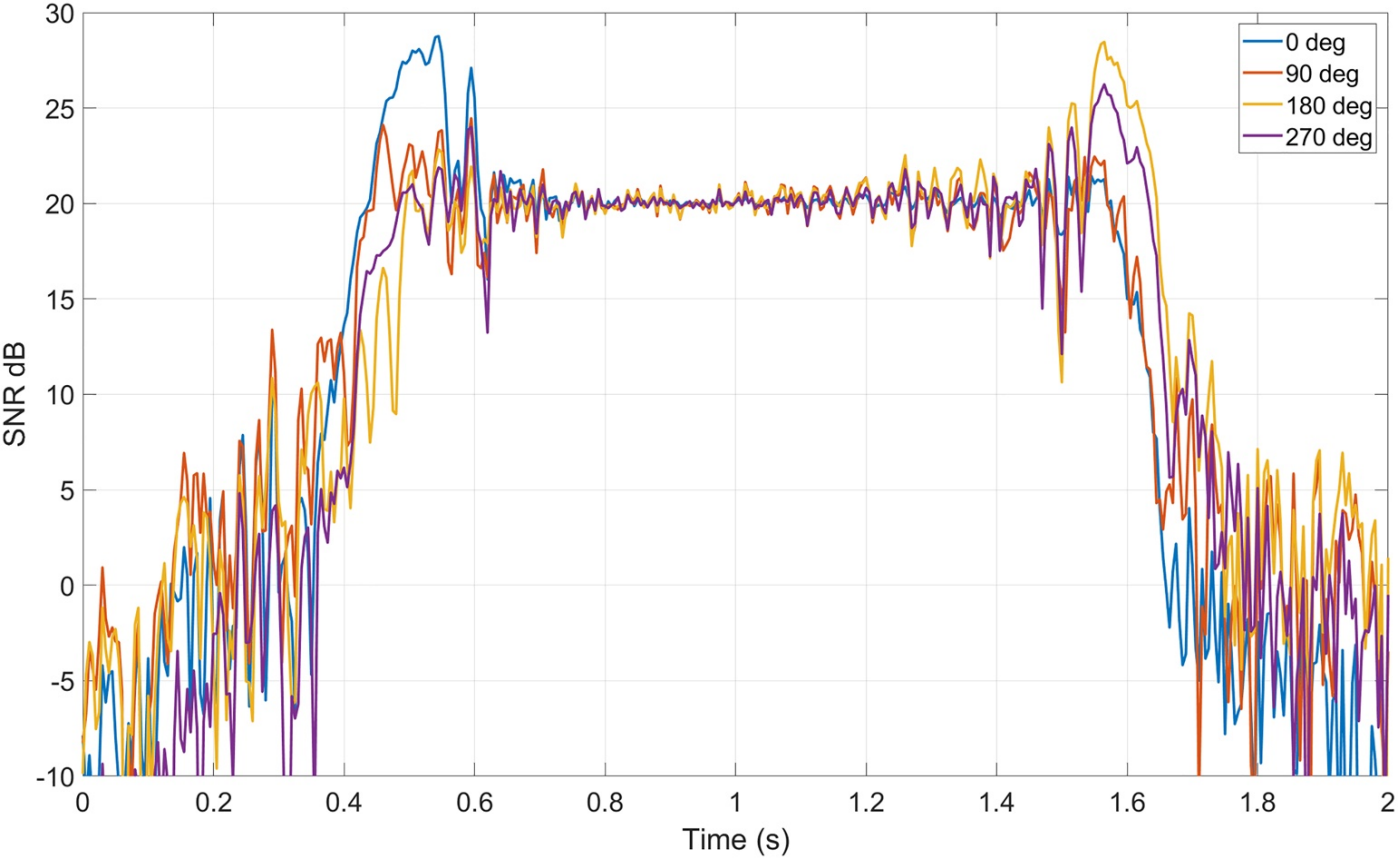
Simulated received power waveforms for four wind directions at 2 m/s wind speed.

The red line in Figure 11 shows an example track we use in this paper. For this track, the incidence angle was 22°, and the specular point travels northeast along the track. Using the coherent scattering model, we simulated the expected peak power of the coherent component of the DDM using a) the shape of the lake and the roughness profile with fixed wind direction and varying wind speed (Figure 12) and b) a fixed wind speed with varying direction (Figure 13). The simulated DDMs were formed using a 5 ms coherent integration time and no incoherent averaging, resulting in a fine sampling along the 2 s track. SNR values were calculated using a fixed value for the noise floor of the receiver. As the wind speed increases and the surface roughens, the reflected power decreases. In addition, the shape of the reflected signal is also sensitive to the wind direction, particularly at the land/water boundaries. However, this signal can be complicated by the edge diffraction effects as the specular point passes over the land water/boundary, as shown in (Camps & Munoz‐Martin, 2020). These ripples in the signal can become very significant and may, for example, affect the solution. Determining the extent to which the wind direction signal is measurable in the presence of these ripples would require many Raw IF measurements, which are currently unavailable and is beyond the scope of our investigation.

**Figure 14:**
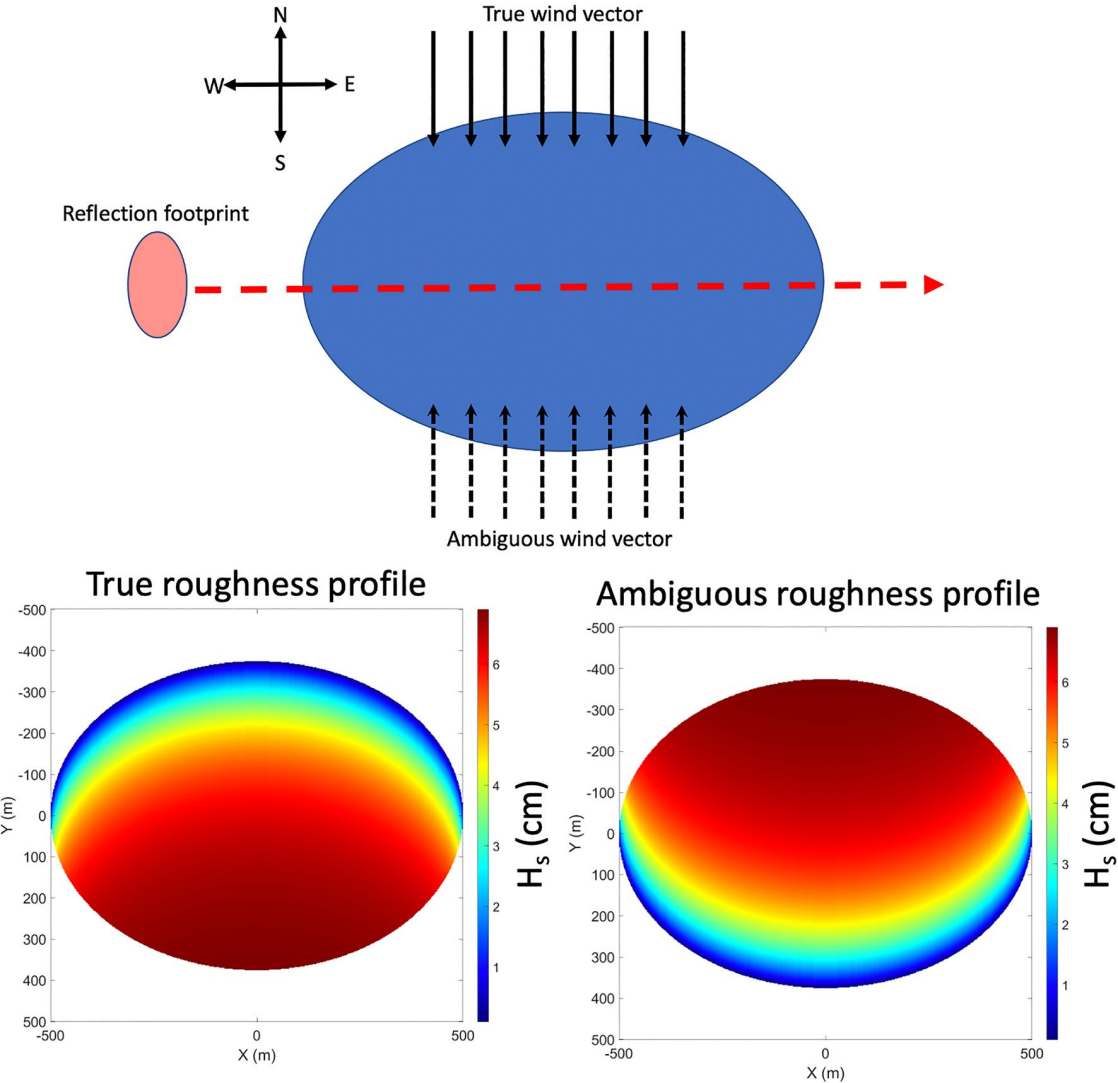
Example reflection scene geometry for an ambiguous power return in the wind direction. The wind speed is set to 2 m/s and the depth was fixed to 1 m. *Note*. top image in figure not to scale.

Another possible complication in a wind vector retrieval is the possibility of ambiguity between wind vectors for select tracks. While the reflected power profiles are highly dependent on the inland water body topography, certain shapes may produce an ambiguous return. One could imagine a scenario where a good fit exists for a number of measured power curves and the modeled power curve. For example, if the specular track were to cross through the center of a water body of symmetric shape, an ambiguity in the wind direction will arise. The top of Figure 14 shows an example of this type of reflection geometry, where the “true” wind is moving from North to South. The “false” solution here occurs in the opposite wind direction (i.e., South to North), as this would produce an identical received power track due to the symmetry in the integration over the surface area. The true surface roughness profile and the ambiguity are shown in the bottom left and right of Figure 14, respectively. It can be seen that the total reflecting surface contributing to the received power at any given point in the specular track will look identical, even though the areas of higher roughness (corresponding to longer fetch) will occur on opposite sides of the water body. The presence of varying wind conditions leading to the same received power curve causes an ambiguity in the received signal. The presence of this ambiguity, similar to the effect of the edge diffraction, is currently not well understood and would require a very large data set in order to better characterize this issue.

The sensitivity of the received power tracks to the wind direction and magnitude provides the possibility to estimate both the surface wave height profile and the associated wind vector. First, a set of power curves for varying wind vectors is generated using the forward model. Then, the residuals are calculated by taking the difference between the measured power curve and the simulated curves. The simulated curve that gives the minimum squared residual is then our estimated surface height profile and wind vector. Because the sensitivity of the received power with wind direction is highly dependent on the shape and size of the water body, it is expected that the retrieval accuracy of the wind magnitude is likely to be greater than the wind direction.

**Figure 15:**
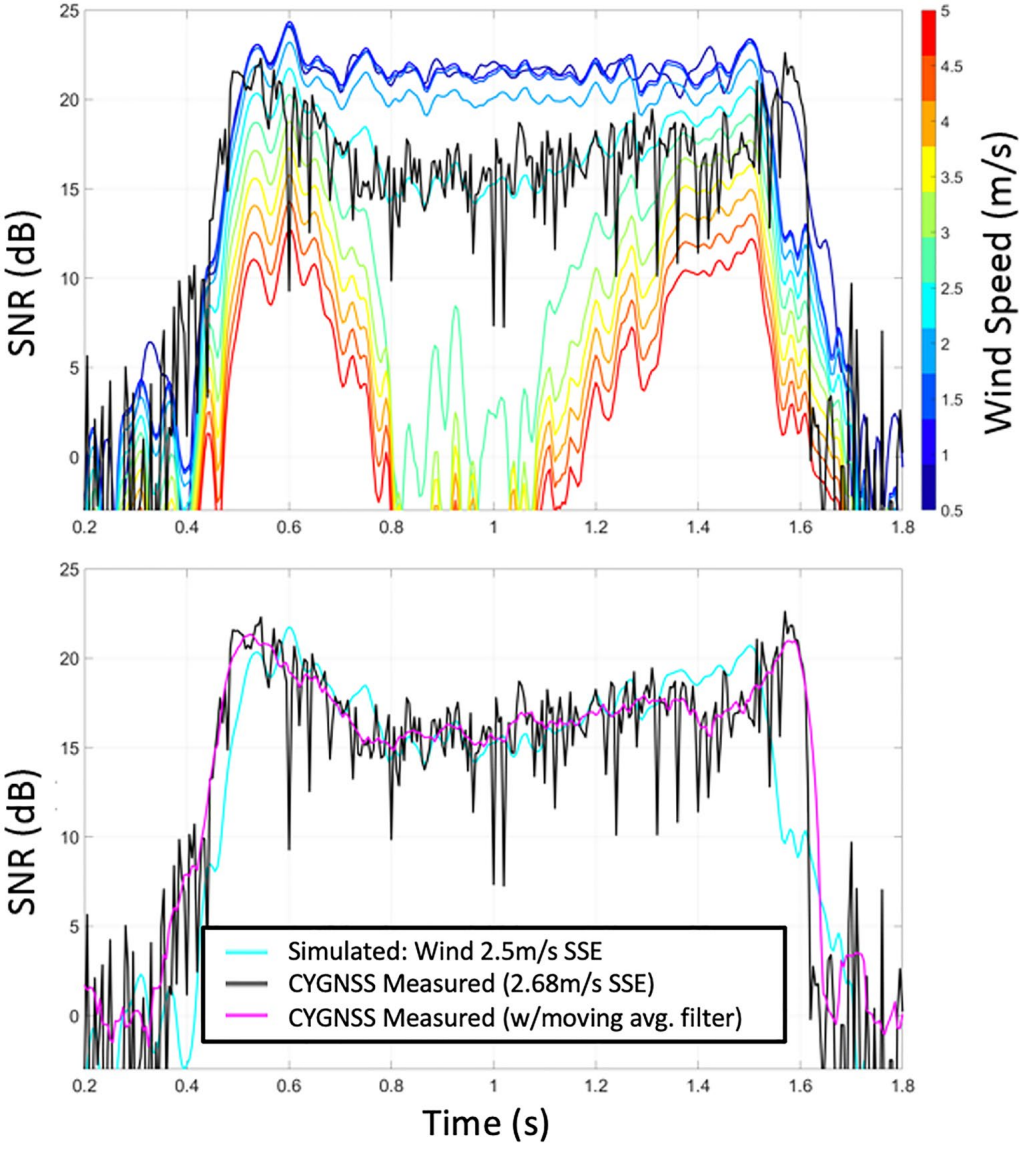
Simulated SNR for different wind speeds with measured CYGNSS data in black (top) and a comparison of measured power to retrieved wind speed curve (bottom).

Figure 15 shows simulated SNR for a range of wind speeds as well as the measured SNR from the CYGNSS raw IF data (shown in black). In this figure, the simulated wind direction was set to S‐SE to match the measured data from the Ilopango Airport weather station several minutes before the track time. The shape of the forward‐modeled power curve generated with 2.5 m/s wind speed matches well the shape of the measured data. The weather station also reported 2.6 ± 0.25 m/s as the measured wind speed near this track time. This agrees well with the best estimate of wind speed from the forward‐model process. Here, we have shown the sensitivity of the signal to the wind speed, and the direction is taken from the local weather station. It is also important to note that this match‐up has an offset between the simulated and measured power. This offset may be caused by the assumption of a specific receiver noise power, the receiver noise figure, limited bandwidth effects, signal quantization loss, uncertainties in the true EIRP or antenna gains, or any other uncertainties in the raw IF data processing chain. For a future system to best utilize the coherent reflection's sensitivity to wind vector, the full link budget and these various effects should be carefully considered.

**Figure 16:**
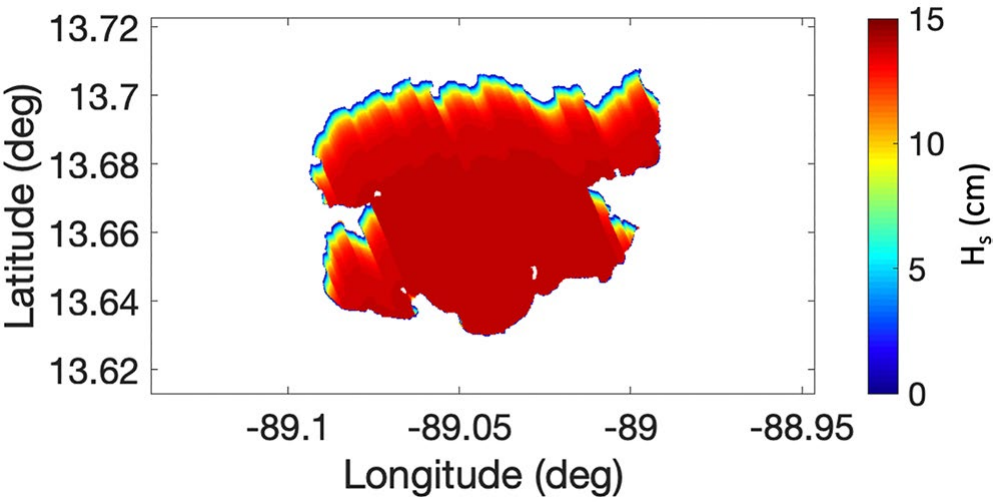
Retrieved *H*_*s*_ profile using the best‐case match‐up procedure with the CERC model.

In addition to the estimation of the wind vector, this method potentially retrieves a surface wave profile. With the selection of a wind speed and direction, the underlying surface wave heights that gave the best fit forward‐modeled power curve is also selected. Figure 16 shows the estimated wave height profile from the model. Unfortunately, validation of the surface wave heights is difficult. At the time of this work, ground truth data sets for the inland water wave heights were unavailable.

### 4.4 Comparing Multiple Observations

The previous section demonstrated successful retrieval of wind speed; however, it was based on a single raw signal CYGNSS recording over the lake. Thus far, a total of 3 recordings have been made from CYGNSS over this lake and are compared below. Details of each track are summarized in Table 1. Note that the wind speed for Tracks #2 and #3 is significantly lower than for Track #1.

**Table 1:** Track Information From CYGNSS Data

	Track #1	Track #2	Track #3
Date (2019)	July 13	Aug 17	Aug 22
Wind Speed (m/s)	2.6	0.8	1.1
Wind Direction	S	N‐NE	SW
Rain Present?	No	Yes	Yes
Inc. Angle (deg)	22	36	15
Ant. Gain (dBi)	10.0	8.7	7.7
GPS PRN Number	11	16	05
CYGNSS FM Number	01	03	07
UTC Track Start Time	12:49:07	04:33:47	17:00:49

For this comparison, GNSS reflections from Lake Ilopango were simulated using the same coherent scattering model presented previously. The local topography was considered when recalculating the specular point track over the lake. No wind speed or wind direction retrieval was performed on these additional tracks. Rather, the point is to highlight the features in all tracks available over the lake.

**Figure 17:**
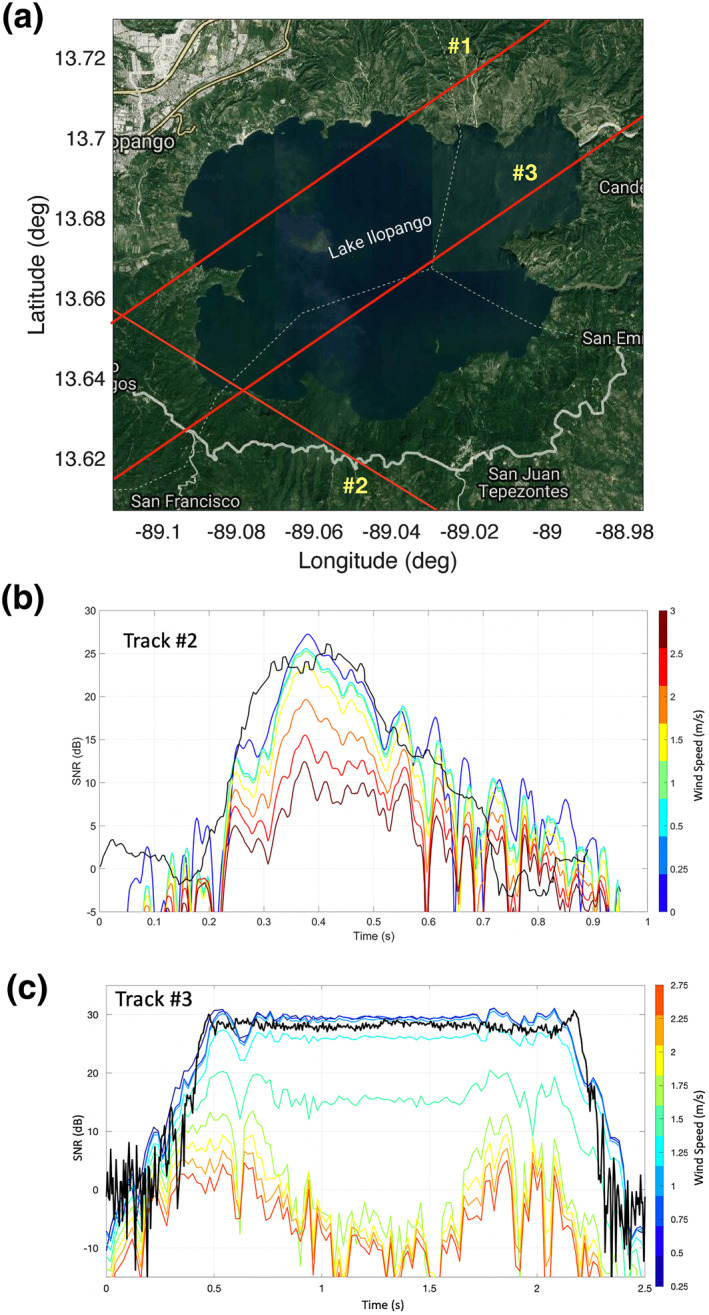
Map of multiple tracks over Lake Ilopango (top) and comparison between the measured (black) and simulated (color) coherent SNR for two additional specular tracks #2 and #3.

Figure 17 shows a comparison between the measured and simulated results for each of the 3 tracks. It should be noted that there are differences in the absolute magnitude of the receiver SNR. Overall uncertainties arise from the true GPS transmitted EIRP, receiver antenna gain, receiver noise power, or other relevant parameters. For the purpose of this work, we do not attempt to retrieve wind speed or magnitude from Tracks #2 and #3 as it would require a precise power budget to estimate wind speed when surface roughness is minimal. These additional cases highlight the limitations of the proposed approach: There is a limited range of wind speeds that can be accurately retrieved.

### 4.5 Non‐Coherent Component of Scattered Power

Due to the surface roughness caused by the wind, a non‐coherent component of the scattered signal also exists alongside the coherent reflection. The strength of the bistatically scattered diffuse signal is dependent on the surface roughness spectrum (Garrison et al., 1998; Zavorotny & Voronovich, 2000). In this section, the relative strength of the diffuse component is compared to the coherent component for the chosen example lake.

**Figure 18:**
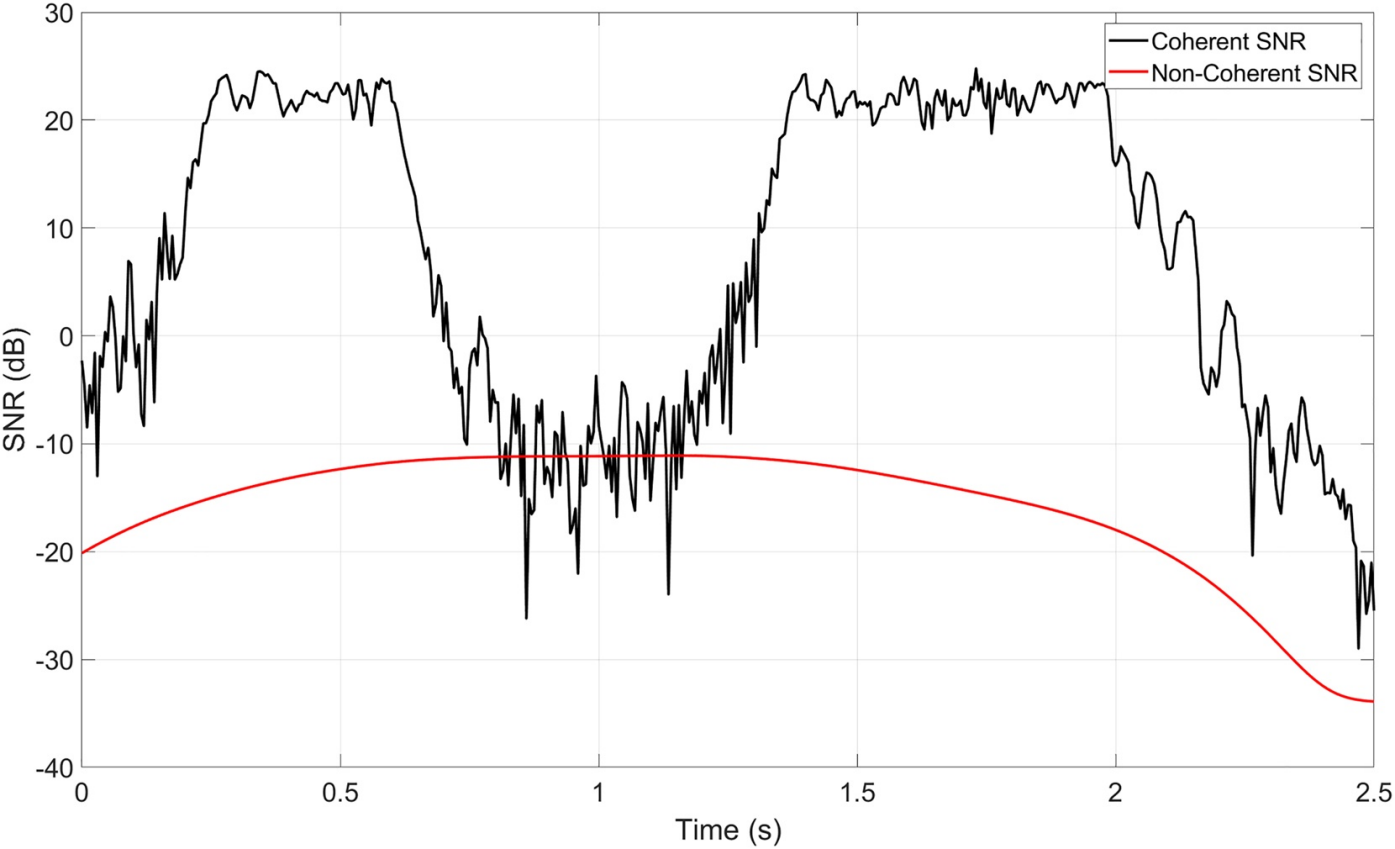
Comparison of simulated non‐coherent scattered power (red) to the simulated coherent power (black) from Lake Ilopango for track #3 with a 2.75 m/s wind speed.

When the roughness is low (as given by a small Rayleigh parameter), the traditional methods of calculating the NBRCS using Geometrical Optics (GO) are inaccurate (Voronovich & Zavorotny, 2017). At low roughness, a higher fidelity scattering model such as the Small Slope Approximation of the first order (SSA1) is used to more accurately capture the surface scattering (Voronovich & Zavorotny, 2018; Voronovich & Zavorotny, 2017; Voronovich, 1999). For this work, SSA1 was implemented using the wind‐generated roughness spectrum from the Elfouhaily model (Elfouhaily et al., 1997). This is an empirical model that is different from the CERC model used previously for the inland water wave heights. Unfortunately, the CERC model only produces estimates of *H*_*s*_, but the SSA1 model requires a roughness spectrum as an input. Resulting values of *H*_*s*_ for lower wind speeds and reduced fetch were found to be very comparable for both the Elfouhaily and the CERC models. Similar to the CERC model, the Elfouhaily model was created for salt–water bodies. However, an examination of the Elfouhaily roughness spectra at various water salinity values (which are associated to the water density (Sharqawy et al., 2010)) found negligible differences between fresh and sea water cases. To compare the relative scales of the power between the coherent and diffuse portions for these small inland water bodies, the approximation of the Elfouhaily roughness model will suffice. NBRCS was calculated for the Track #3 over the surface of Lake Ilopango. Figure 18 shows the simulated received scattered power from this track assuming a 2.75 m/s wind speed and a Southward wind direction. It can be seen here that the relative contribution to the total power from the diffuse scattering is quite low, and the resulting SNR is low. In this case, the SNR due to the roughened water surface is low because not only the wind speed is low but also because Lake Ilopango itself is a relatively small surface as compared to the non‐coherent footprint set by the WAF. The total received non‐coherent power scales with area (within the limits of the WAF footprint). Therefore, the total non‐coherent reflected power from small water bodies will also be low given the small surface area.

**Figure 19:**
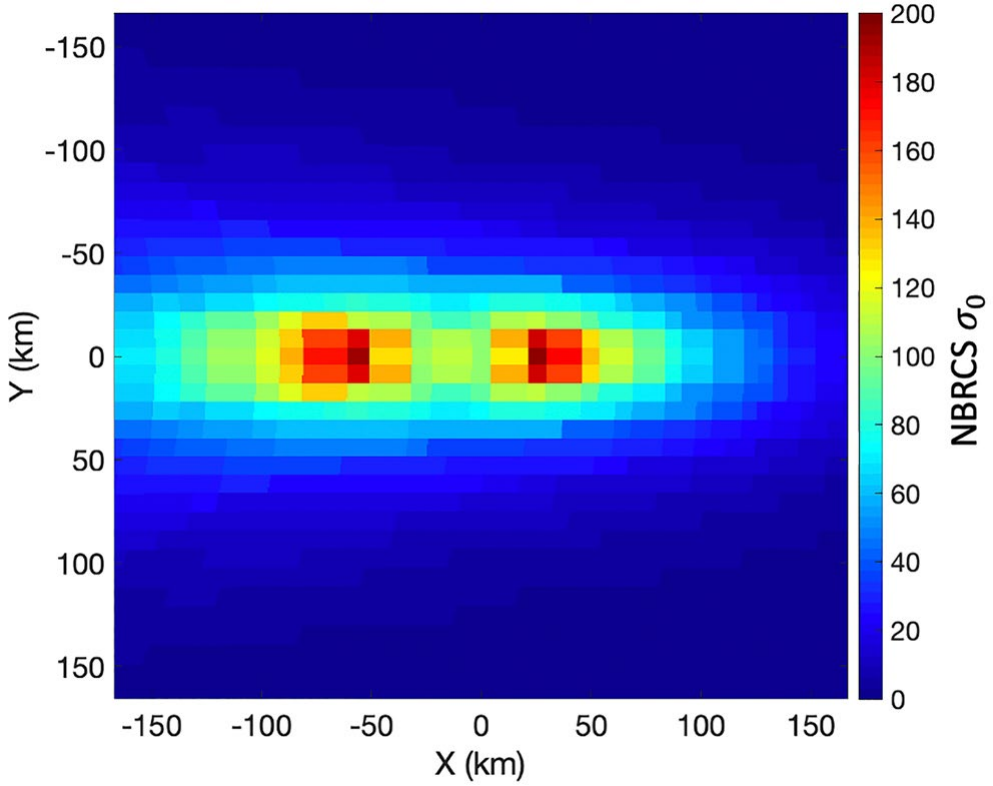
Map of NBRCS values across reflecting surface area calculated using SSA1 at 2.5 m/s wind speed (*H*_*s*_ = 14 cm) with Elfouhaily surface spectrum model for CYGNSS standard scattering geometry at 45° incidence angle.

For larger inland water bodies, the diffuse component will be more significant since the scattering area is larger and larger waves will be supported. To quantify when the non‐coherent component will be significant, the coherent and non‐coherent contributions to the total received power for increasing values of *H*_*s*_ will be calculated for a generic scenario. Here, a homogeneous, infinite plane surface is considered. Using a CYGNSS reflection geometry with an incidence angle of 45° and *T*_*c*_ = 1 ms, the received power is calculated for increasing significant wave heights. The diffuse power is calculated through the NBRCS across the surface using SSA1. A constant receiver antenna gain pattern (*G*_*r*_) across the surface is assumed. The calculated *σ*_0_ across the surface using SSA1 is shown in Figure 19. This shows the interesting case where the NBRCS has a bi‐modal distribution across the surface. This result for a sufficiently low wind speed and fetch is fully explained in (Voronovich & Zavorotny, 2017).

**Figure 20:**
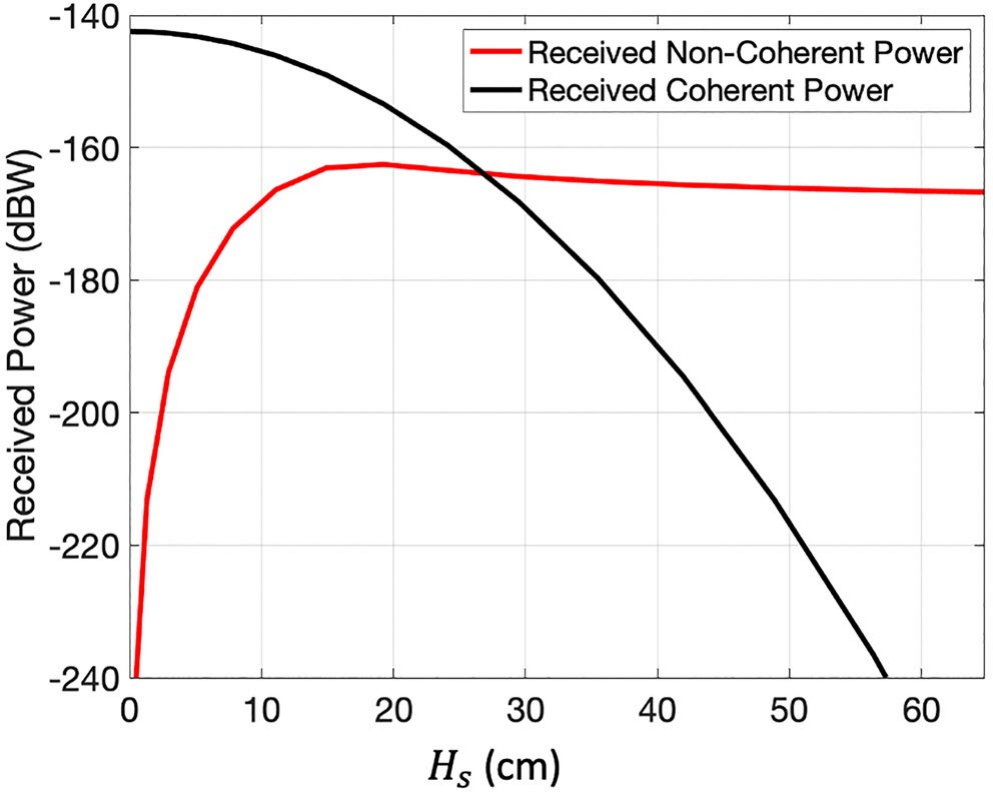
Comparison of non‐coherent scattered power (red) to the coherent power (black) in the DDM specular bin for an infinite homogeneous scene.

Figure 20 shows the comparison between the coherent and non‐coherent components for this scene. The received power, taken from the delay‐Doppler bin, which contains the specular point, is used to evaluate the SNR. It can be observed that the cross‐over point where the diffuse power becomes greater than the coherent power occurs near a *H*_*s*_ of 27 cm. As expected through the attenuation factor *ψ*, the coherent power reduces quickly with increasing *H*_*s*_. Also of note, the diffuse power increases quickly with *H*_*s*_, reaches a peak, then slowly reduces. In addition, at low wave heights, the coherent power is several orders of magnitude greater than the diffuse power, even at the diffuse power's peak value.

Since we are using an infinite surface, the total surface area contributing to the diffuse power in this scene is constrained by the size of the WAF. If we consider reflections from smaller inland water bodies than the size of the WAF (such as the previous example in Figure 18), the amount of non‐coherent power contributed from the water body will be reduced as well. There may also be non‐coherent scattering from the land areas, which is not considered here and the relative contribution from surrounding land area will depend on the amount of land within the WAF footprint and its surface properties.

## 5 Summary and Conclusions

The method described in this paper indicates that information about the wind magnitude and direction is contained within the shape of the measured signal as a specular point transects an inland water body, almost independently of the absolute magnitude. It was shown that the coherent signal is highly sensitive to roughness variations over the water surface. The surface roughness depends on the wind‐driven wave heights, which vary predictably over the surface for different wind speeds and wind directions. It has been observed that the CYGNSS Level‐1 peak SNR data over an inland water body such as Lake Ilopango exhibits clear dependence on wind vector. The forward model for the scattered power in combination with a wave model demonstrates potential for the retrieval of wave heights and wind vector. This new source of wind vector data is very valuable to investigate the lake surface heat balance, in particular for cloudy/rainy conditions and non‐instrumented areas. The CYGNSS raw signal data were used to validate a retrieval of wind speed using a mean‐squared error minimization approach with promising results. The scattering effects of the lake edges and/or the possible ambiguity given by the geometry likely complicate the retrieval of wind vector. Finally, we have shown evidence that the coherent component of the reflection from small inland waters is dominant as compared to the non‐coherent scattering. However, if the water body is large, non‐coherent reflection becomes the dominant scattering mechanism when the surface roughness increases.

Results in this paper were based on a simple example lake with no vegetation. In such cases, the interaction between the wind and the water surface can be described by wave models, and these models work well in unprotected inland waters. However, vegetated inland waters such as wetlands and marshes are of significant scientific interest, and vegetation will have a significant influence on the final surface roughness. Work is underway to identify and incorporate several vegetation effects on inland surface water roughness into our GNSS‐R coherent scattering model.

## Data Availability

CYGNSS Level‐1 data are available from the Physical Oceanography DAAC (CYGNSS. 2018. CYGNSS Level 1 Science Data Record Version 2.1. Ver. 2.1. PO. DAAC, CA, USA. Data set accessed [2020‐10‐23] at https://doi.org/10.5067/CYGNS-L1X21). This work uses the “DDM SNR” variable over the year 2017. Wind speed and direction data available from the NOAA Integrated Surface Database (NOAA National Centers for Environmental Information (2001): Global Surface Hourly [01/01/2017–09/01/2019]. NOAA National Centers for Environmental Information [10/23/2020]). Selected variables are “wind speed” and “wind direction” and “precipitation.” CYGNSS Raw IF data provided at (CYGNSS. 2020. CYGNSS Level 1 Raw Intermediate Frequency Data Record. Ver. 1.0. PO. DAAC, CA, USA. Data set accessed [2020‐10‐23] at https://doi.org/10.5067/CYGNS-L1RIF).
